# Evaluation of QTc interval prolongation in patients with advanced clear cell renal cell carcinoma treated with first line sunitinib

**DOI:** 10.1007/s00280-026-04884-y

**Published:** 2026-04-29

**Authors:** Agata Sałek-Zań, Mirosława Püsküllüoğlu, Agnieszka Stąpór, Justyna Jaworska, Agnieszka Pietruszka, Aleksandra Grela-Wojewoda, Maciej Stąpór, Tomasz Banaś

**Affiliations:** 1 Department of Clinical Oncology, Kraków Branch, Maria Sklodowska-Curie National Research Institute of Oncology, Garncarska 11, Kraków, 31-115 Poland; 2 Faculty of Medicine, Institute of Cardiology, Department of Interventional Cardiology, Jagiellonian University Medical College, St. John Paul II Hospital, Kraków, Poland; 3 Department of Radiation Oncology, Kraków Branch, Maria Sklodowska-Curie National Research Institute of Oncology, Kraków, Poland

**Keywords:** Sunitinib, Cardiotoxicity, QTc prolongation, Renal cell carcinoma

## Abstract

**Background:**

Sunitinib, a multitargeted tyrosine kinase inhibitor, is used in advanced clear cell renal cell carcinoma (ccRCC). Corrected QT interval (QTc) prolongation is a recognized cardiovascular toxicity of this therapy.

**Aim:**

To evaluate the incidence, timing, and severity of QTc prolongation in real-world advanced ccRCC patients treated with first-line sunitinib, compare Bazett’s and Fridericia’s correction formulas, and identify risk factors.

**Materials and methods:**

This retrospective single-center cohort study included 67 patients between January 2019 and June 2022. QTc was calculated manually using Bazett’s and Fridericia’s formulas and graded per Common Terminology Criteria for Adverse Events (CTCAE) v5.0. The incidence, timing, and severity of QTc prolongation were assessed based on the highest recorded QTc value. Statistical analyses compared QTc values between formulas and evaluated potential clinical predictors of QTc prolongation.

**Results:**

The median treatment duration was 1.4 years (IQR 0.72–2.99). QTc prolongation ≥ 450ms occurred in 44.8% of patients (Bazett) and 34.3% (Fridericia) and ≥ 500ms in 7 and 6 patients, respectively. The median onset of QTc ≥ 450ms was cycle 7.9 (Bazett) and 10.4 (Fridericia). Bazett’s values were significantly higher across most treatment cycles (*p* < 0.05). Older age, thyroid dysfunction, smoking history, hypertension and soft-tissue metastases were associated with increased risk of QTc prolongation.

**Conclusions:**

QTc prolongation was frequent and occurred both early and later during therapy. Fridericia’s formula provided more accurate and stable values and should be preferred for monitoring. Repeated ECG measurements and close cardio-oncology collaboration are essential.

**Supplementary Information:**

The online version contains supplementary material available at https://doi.org/10.1007/s00280-026-04884-y.

## Introduction

Kidney cancer in adults is most commonly represented by renal cell carcinoma (RCC), with clear cell RCC (ccRCC) accounting for about three-quarters of cases. While surgery is effective for localized disease, many patients present with metastases or locally advanced tumors. Advances in systemic therapy, including tyrosine kinase inhibitors (TKIs) and immune checkpoint inhibitors (ICIs), have improved outcomes in advanced ccRCC and current algorithms emphasize risk-adapted, molecularly informed strategies [1–3]. Despite the widespread adoption of ICI-based combinations, no significant overall survival (OS) benefit over sunitinib, an oral multitargeted TKI, has been demonstrated in favorable-risk patients with long-term follow-up [4–6]. Sunitinib remains relevant first-line treatment for patients ineligible for immunotherapy [1, 2, 7].

The QT interval reflects the time taken for ventricular repolarization. Its prolongation is associated with an increased risk of cardiac arrhythmias, including torsades de pointes, which may ultimately result in ventricular fibrillation. This can lead to sudden cardiac death [8]. Since the QT interval shortens with faster heart rates (HR), the corrected QT interval (QTc) serves as a predictor of pro-arrhythmic risk [9]. The elongation of the QT interval is influenced by age, gender, time of day, autonomic nervous system activity, electrolyte imbalances, cardiovascular diseases (CVD), liver and kidney disorders, hypothyroidism, diabetes and diet [10–12]. Certain medications prolong QT, an effect intensified by drug interactions QTc interval prolongation is a clinically significant adverse effect (AE) observed with various anticancer therapies [11–14].

The anticancer activity of TKIs involves blocking the abnormal activation of cellular signalling pathways [15]. In TKIs, QTc prolongation is mainly caused by blockade of the rapid component of the delayed rectifier potassium current (IKr) [10, 12]. Patient with ccRCC, are predisposed to QTc prolongation because of multiple overlapping risk factors: prior cardiotoxic treatments, baseline cardiovascular comorbidities, electrolyte disturbances and polypharmacy [12, 16, 17]. Sunitinib has been identified as a targeted anticancer agent associated with a dose-dependent, clinically meaningful risk of QTc prolongation [12, 17–19]. Routine ECG monitoring is not mandated in the Sunitinib Summary of Product Characteristics (SmPC). However, ECG is advised in patients with QT prolongation history, QT-prolonging drugs, CYP3A4 inhibitors, electrolyte disturbances, bradycardia or cardiac comorbidities [10, 14, 19].

The study aimed to determine the incidence of QTc prolongation in real-world ccRCC patients treated with first-line sunitinib, assess differences between correction formulas, and identify clinical risk factors for its occurrence.

## Materials and methods

### Study design and patient population

This retrospective, single-center study included all adult patients with histologically confirmed ccRCC who started first-line palliative sunitinib between January 2019 and June 2022 and received at least one full cycle (minimum 6 weeks of treatment). According to SmPC, sunitinib dosing was 50 mg daily for 28 days with a 14-day break (4/2 dosing schedule), reduced to 37 mg or 25 mg if AEs occurred [19]. Inclusion further required the availability of interpretable ECG at baseline and during the treatment as required by the national reimbursement program. The presence of a pacemaker constituted an exclusion criterion. All clinical and ECG data were extracted retrospectively from electronic medical records and verified by two independent reviewers.

### Clinical data

The following variables were collected: age, sex, body mass index (BMI), performance status according to the Karnofsky scale, Memorial Sloan Kettering Cancer Center (MSKCC) and International Metastatic RCC Database Consortium (IMDC) prognostic scores [20–22], time from diagnosis to treatment initiation and site(s) of metastatic involvement. Treatment-related data included sunitinib dose, duration of therapy, best response achieved and treatment-related AEs.

Laboratory values, including assessment of serum electrolyte levels and thyroid function parameters were checked before each ECG. If abnormalities were detected, treatment was initiated according to appropriate guidelines. The patient was also interviewed about her medication use.

### Electrocardiographic assessment and definitions

ECGs were performed as part of routine care and retrieved from institutional digital archives, using the certified AsCARD MrBlue – Aspel device. ECGs were performed at baseline and subsequently at routine cycle-start visits (every 6 weeks), usually in the morning, before drug administration, i.e., after the planned off-treatment interval.

When an ECG could not be used for analysis (e.g., poor quality or excluded due to fever/infection), that time point was omitted from serial analyses; patients remained included if they had baseline and at least one interpretable on-treatment ECG.

Measurements of QT and RR intervals were manually assessed by 2 independent researchers according to the same criteria. The QT interval was determined from the start of the QRS complex to the conclusion of the T wave. All 12 ECG leads were evaluated, but manual measurements of QT interval were taken from leads II or V5 (depending on availability) for further statistical analysis. We used maximum slope intercept method to define the end of T waves. U waves < 0.1 mV in amplitude or independent from T wave were excluded from the QT measurement. When U waves were larger (> 2 mV) and merged with the T wave they were included in the QT measurement.

Corrected QT intervals (QTc) were calculated using both Bazett’s formula (QTcB = QT/√RR) and Fridericia’s formula (QTcF = QT / RR^1/3) [8]. All calculations QTc, also in patients with persistent atrial fibrillation, have been carried out in accordance with the guidelines of the European Society of Cardiology [23, 24]. In situations where the results of a given ECG analysis were different, the evaluators met and jointly reviewed the recording, after which a consensus was reached regarding the result.

The severity of QTc prolongation was graded according to version 5.0 of the Common Terminology Criteria for Adverse Events (CTCAE): grade 1 (QTc 450–480 ms), grade 2 (QTc 481–500 ms), grade 3 (QTc > 500 ms or an increase of > 60 ms from baseline), and grade 4 (life-threatening arrhythmia, including torsade de pointes) [25].

For each patient, the longest QTc interval recorded during the course of sunitinib treatment was considered for grading. Serial ECGs were analyzed to assess temporal trends in QTc values. Baseline QTc was defined as the value obtained from the earliest ECG performed prior to sunitinib initiation, typically on day 1 of the first treatment cycle. The timing of QTc prolongation was defined as the first treatment cycle in which the QTc interval exceeded the specified threshold, as measured by either correction method.

Treatment discontinuation due to QTc prolongation was defined as permanent cessation of sunitinib therapy directly attributed to QTc abnormalities. Treatment continuation despite QTc prolongation referred to the maintenance of sunitinib therapy following documentation of QTc values above the defined thresholds.

### Ethical considerations

The study was approved by the institutional ethics committee of the Maria Sklodowska-Curie National Research Institute of Oncology, Warsaw Branch (registry no. 6/23, October 5, 2023). All procedures followed institutional, national, and Helsinki Declaration standards. Due to the retrospective design and anonymized data, individual consent was not required.

### Statistical analysis

All statistical analyses were performed using R software, version 4.5.1 [26]. Descriptive statistics summarized the study population. Continuous variables were presented as means with standard deviations (SD), medians with interquartile ranges (IQR), and full ranges, depending on data distribution. Categorical variables were presented as frequencies and percentages.

QTc intervals calculated using Bazett’s and Fridericia’s formulas were compared with the Wilcoxon signed-rank test across multiple ECG time points; a two-sided p-value < 0.05 was considered significant. QTc prolongation frequency and severity were based on the highest recorded QTc during treatment. For both formulas, the number of patients exceeding clinically relevant thresholds and the treatment cycle of first occurrence were recorded, including mean, minimum, and maximum cycle numbers to assess temporal patterns. The proportion of patients continuing or discontinuing treatment after QTc elongation was calculated separately for each threshold and correction method. Univariate logistic regression identified clinical factors associated with QTc prolongation, using separate models for grade ≥ 1, ≥2, and ≥ 3 events. Odds ratios (ORs) along with their 95% confidence intervals (CIs) and p-values were presented. Due to limited sample size, multivariable analysis was not performed. Per-cycle paired tests were used as an exploratory within-visit comparison between formulas. No adjustment for multiple comparisons across cycles was applied; therefore, p-values should be interpreted descriptively.

## Results

### Patient characteristics

Among 96 patients with RCC who initiated first-line palliative treatment with sunitinib at the study center, 67 individuals met the inclusion criteria and were included in the final analysis. The median age at cancer diagnosis was 61 years (interquartile range: 52.5–66.0; range: 40–79 years). The cohort included 25 females (37.31%) and 42 males (62.69%). Further details are presented in Table 1.

**Table 1:** Patients characteristic

Parameter		*N* = 67
Age at the start of sunitinib treatment (years)	Median (interquartile range) Range	63 (57-69.5) 45–83
The time from the diagnosis of kidney cancer to the start of sunitinib treatment (years)	Median (interquartile range) Range	1.21 (0.37–4.31) 0.08–17.02
Karnofsky score at the start of sunitinib treatment	100 90 80	17 (25.37%) 41 (61.19%) 9 (13.43%)
Body mass index at the start of sunitinib treatment (kg/m²)	Median (interquartile range) Range	26.79 (24.8-29.41) 19.23–42.22
Smoking history	No Yes	38 (56.72%) 29 (43.28%)
MSKCC scale	MSKCC 0 MSKCC 1 MSKCC 2	27 (40.30%) 25 (37.31%) 15 (22.39%)
IMDC scale	IMDC 0 IMDC 1 IMDC 2 IMDC 3 IMDC 4	24 (35.82%) 24 (35.82%) 11 (16.42%) 7 (10.45%) 1 (1.49%)
Metastatic site*	Adrenal glands Bones Brain Kidneys Liver Lungs Lymph nodes Ovaries Pancreas Peritoneum Soft tissues Thyroid	13 (19.40%) 16 (23.88%) 2 (2.99%) 5 (7.46%) 7 (10.45%) 38 (56.72%) 25 (37.31%) 1 (1.49%) 6 (8.96%) 9 (13.43%) 25 (37.31%) 2 (2.99%)
Comorbidities*	Any comorbidities Asthma Cataract Chronic kidney disease Chronic obstructive pulmonary disease Depression Diabetes mellitus Epilepsia Gastritis Glaucoma Gout Heart failure / Myocardial infarction Heart rhythm disorders Heart valve disorders Hepatic steatosis Hypertension Hypercholesterolemia / Hyperlipidemia Hyperthyroidism Hypothyroidism Intestinal dieases Ischemic heart disease Neurotic disorders Obesity Ostreoarthritis Other neoplasm Pancreatis Positive hepatitis B surface antigen Prostatic hyperplasia Psoriasis Rheumatoid arthritis Thromboembolic diseases Uterine fibroid Vascular diseases	61 (91.04%) 1 (1.49%) 1 (1.49%) 1 (1.49%) 5 (7.46%) 3 (4.48%) 17 (25.37%) 2 (2.99%) 3 (4.48%) 3 (4.48%) 2 (2.99%) 6 (8.96%) 6 (8.96%) 2 (2.99%) 2 (2.99%) 47 (70.15%) 13 (19.40%) 4 (5.97%) 9 (13.43%) 2 (2.99%) 11 (16.42%) 1 (1.49%) 4 (5.97%) 10 (14.93%) 1 (1.49%) 1 (1.49%) 1 (1.49%) 6 (8.96%) 1 (1.49%) 1 (1.49%) 4 (5.97%) 1 (1.49%) 15 (22.39%)

*Multiple choice question - percents do not sum up to 100

Abbreviations: IMDC, International Metastatic RCC Database Consortium; MSKCC, Memorial Sloan Kettering Cancer Center

### Treatment exposure, safety and clinical outcomes

The mean duration of drug exposure was 2.18 years (SD 2.26), with a median of 1.4 years (IQR 0.72–2.99; range 0.31–11.89). The most commonly reported AEs were gastrointestinal (73.13%), dermatological and cardiac (53.73%), endocrinological (40.30%) and hematological (38.81%) and urological (26.87%).

In our cohort, 53.7% (36 of 67) patients encountered cardiovascular adverse events. The most frequent event was hypertension (77.8%, 28/36), followed by rhythm/conduction disorders (22.2%, 8/36), thromboembolic events (13.9%, 5/36) and heart failure with reduced ejection fraction (11.1%, 4/36). The simultaneous occurrence of two or more distinct cardiovascular adverse events was observed in 19.4% (7 out of 36) of cases. No arrhythmia-related hospitalizations, torsade de pointes, ventricular tachyarrhythmias or sudden cardiac death were recorded.

We assessed whether the severity of gastrointestinal adverse events (GI AEs) -graded according to CTCAE; ≥G2/≥G3, was associated with QTc prolongation (G1–G3) calculated by Bazett’s and Fridericia’s formulas. In univariable logistic regression models, GI AE severity was not a significant predictor of QTc prolongation across any grade (all *p* > 0.05).

Adjustments to treatment prompted by adverse events were observed in 65.67% of cases with delays, 50.75% involving at least one dose reduction, 34.33% experiencing two dose reductions, and 10.45% leading to permanent discontinuation due to toxicity.

We explored whether QTc prolongation occurring at any time during treatment was associated with subsequent treatment delivery modifications, such as delays, dose reductions, and toxicity-related discontinuation: Tables 2 and 3. Additionally, we assessed whether QTc prolongation was less frequent among patients who required dose reductions. As shown in Tables 2 and 3, the frequency of QTc prolongation did not differ significantly between patients with at least one dose reduction and those without (all p values > 0.05 across grades). Therefore, we did not observe a lower occurrence of QTc prolongation in the dose-reduction group in this analysis. It is important to note that this comparison is descriptive and does not establish causality, as dose reductions were driven by overall toxicity and the timing of QTc prolongation was not modelled.

**Table 2:** The impact of the occurrence of individual degrees of QTc prolongation according to Bazett’s formula on the course of treatment

Parameter		Bazett’s G1		p	Bazett’s G2		p	Bazett’s G3		p
		No (*N* = 37)	Yes (*N* = 30)		No (*N* = 52)	Yes (*N* = 15)		No (*N* = 60)	Yes (*N* = 7)	
Treatment delays	No	15 (40.54%)	8 (26.67%)	*p* = 0.352	20 (38.46%)	3 (20.00%)	*p* = 0.309	21 (35.00%)	2 (28.57%)	*p* = 1
	Yes	22 (59.46%)	22 (73.33%)		32 (61.54%)	12 (80.00%)		39 (65.00%)	5 (71.43%)	
Dose reduction (at least one)	No	19 (51.35%)	14 (46.67%)	*P* = 0.892	28 (53.85%)	5 (33.33%)	*p* = 0.268	30 (50.00%)	3 (42.86%)	*p* = 1
	Yes	18 (48.65%)	16 (53.33%)		24 (46.15%)	10 (66.67%)		30 (50.00%)	4 (57.14%)	
2 dose reductions	No	27 (72.97%)	17 (56.67%)	*p* = 0.255	39 (75.00%)	5 (33.33%)	*p* = 0.007*	43 (71.67%)	1 (14.29%)	*p* = 0.005*
	Yes	10 (27.03%)	13 (43.33%)		13 (25.00%)	10 (66.67%)		17 (28.33%)	6 (85.71%)	
Discontinuation of treatment due to toxicity	No	33 (89.19%)	27 (90.00%)	*p* = 1	46 (88.46%)	14 (93.33%)	*p* = 1	54 (90.00%)	6 (85.71%)	*p* = 0.556
	Yes	4 (10.81%)	3 (10.00%)		6 (11.54%)	1 (6.67%)		6 (10.00%)	1 (14.29%)	
Drug exposure [years]	MEAN (SD)	1.58 (1.97)	2.91 (2.4)	*p* = 0.005*	1.66 (1.87)	3.96 (2.63)	*p* < 0.001*	1.75 (1.82)	5.83 (2.47)	*p* < 0.001*
	Median (quartile)	1.11 (0.67–1.57)	2.42 (1.03–4.04)		1.15 (0.66–1.8)	3.95 (2.28–4.86)		1.16 (0.7–2.22)	5.03 (4.18–7.38)	
	Range	0.31–11.89	0.33–9.63		0.31–11.89	0.33–9.63		0.31–11.89	2.99–9.63	
	n	30	37		52	15		60	7	

p - Qualitative variables: chi-squared or Fisher’s exact test. Quantitative variables: Mann-Whitney test, *statistically significant (*p* < 0.05)

**Table 3:** The impact of the occurrence of individual degrees of QTc prolongation according to Fredericia’s formula on the course of treatment

Parameter		Fridericia’sG1		p	Fridericia’s G2		p	Fridericia’s G3		p
		No (*N* = 44)	Yes (*N* = 23)		No (*N* = 55)	Yes (*N* = 12)		No (*N* = 61)	Yes (*N* = 6)	
Treatment delays	No	17 (38.64%)	6 (26.09%)	*p* = 0.449	21 (38.18%)	2 (16.67%)	*p* = 0.195	22 (36.07%)	1 (16.67%)	*p* = 0.656
	Yes	27 (61.36%)	17 (73.91%)		34 (61.82%)	10 (83.33%)		39 (63.93%)	5 (83.33%)	
Dose reduction (at least one)	No	25 (56.82%)	8 (34.78%)	*P* = 0.145	30 (54.55%)	3 (25.00%)	*p* = 0.124	32 (52.46%)	1 (16.67%)	*p* = 0.197
	Yes	19 (43.18%)	15 (65.22%)		25 (45.45%)	9 (75%)		29 (47.54%)	5 (83.33%)	
2 dose reductions	No	33 (75.00%)	11 (47.83%)	*p* = 0.051	42 (76.36%)	2 (16.67%)	*p* < 0.001*	44 (72.13%)	0 (0.00%)	*p* = 0.001*
	Yes	11 (25.00%)	12 (52.17%)		13 (23.64%)	10 (83.33%)		17 (27.87%(	6 (100%)	
Discontinuation of treatment due to toxicity	No	39 (88.64%)	21 (91.30%)	*p* = 1	50 (90.91%)	10 (83.33%)	*p* = 0.6	54 (88.52%)	6 (100%)	*p* = 1
	Yes	5 (11.36%)	2 (8.70%)		5 (9.09%)	2 (16.67%)		7 (11.48%)	0 (0.00%)	
Drug exposure [years]	MEAN (SD)	1.56 (1.86)	3.36 (2.51)	*P* < 0.001*	1.65 (1.82)	4.6 (2.53)	*p* < 0.001*	1.83 (1.9)	5.72 (2.75)	*p* = 0.001*
	Median (quartile)	1.09 (0.61–1.6)	2.57 (1.5–4.49)		1.15 (0.65–1.85)	4.18 (2.86–5.3)		1.17 (0.71–2.35)	4.55 (3.98–7.75)	
	Range	0.31–11.89	0.33–9.63		0.31–11.89	0.33–9.63		0.31–11.89	2.99–9.63	
	n	44	23		55	12		61	6	

p - Qualitative variables: chi-squared or Fisher’s exact test. Quantitative variables: Mann-Whitney test

*Statistically significant (*p* < 0.05)

The most frequent response to treatment was stable disease, which was seen in 58.21% of patients.

### Frequency and severity of QTc prolongation

The mean QTc interval at baseline was 406.11 ms (SD 47.44) using Bazett’s formula and 395.05 ms (SD 45.12) using Fridericia’s formula. Bazett-derived QTc values were generally higher than Fridericia-derived values at routine assessments, with statistically significant differences observed at most early and mind-treatment measurement points (consecutive cycles of therapy), beginning at baseline and persisting across multiple treatment cycles (Supplementary materials, Table S1). Although Supplementary Table S1 is truncated at cycle 40 due to attrition, QTc prolongation events were recorded throughout treatment, with rare late occurrences observed in individual patients (up to cycles 57 and 73). No arrhythmic events or arrhythmia-related hospitalizations were recorded.

**Fig. 1:**
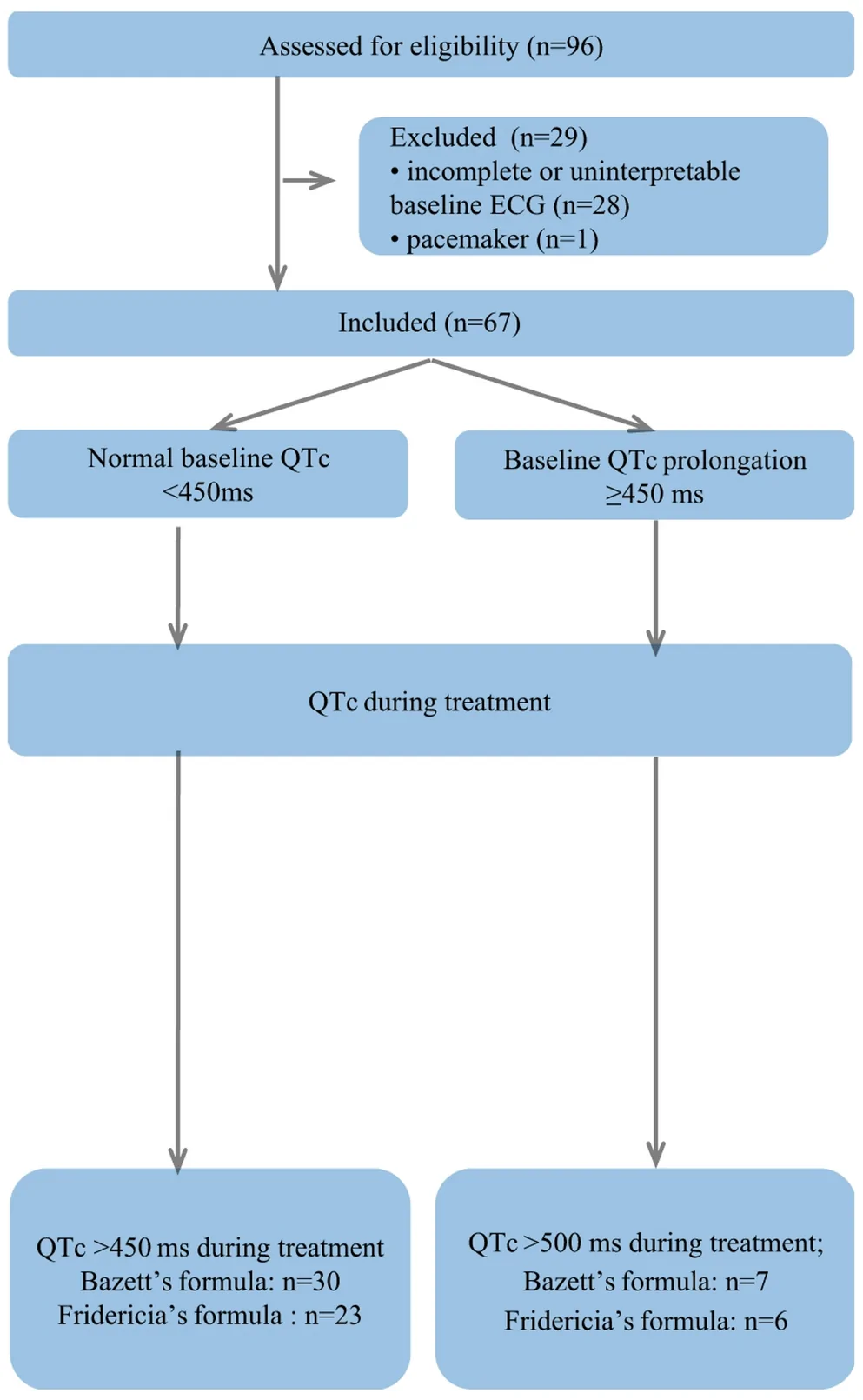
Study flow and QTc assessment at baseline and during treatment

At baseline, QTc > 450 ms was recorded in 15 patients (Bazett) and 7 patients (Fridericia), while QTc > 480 ms occurred in 4 patients (Bazett) and 2 patients (Fridericia). During treatment, QTc > 450 ms was observed in 30 patients (Bazett) and 23 patients (Fridericia); QTc > 500 ms was noted in 7 (Bazett) and 6 patients (Fridericia). Figure 1 illustrates the study flow chart.

The mean cycle in which QTc exceeded 450 ms was cycle 7.90 (Bazett) and 10.39 (Fridericia); for 500 ms, cycle 24.14 (Bazett) and 23.17 (Fridericia) – details in Table 4.

**Table 4:** Timing of QTc interval threshold exceedance during treatment according to Bazett’s and Fridericia’s formulas

Formula	Threshold	*N*	Mean cycle (weeks) with threshold exceeded	Minimum cycle (weeks) with threshold exceeded	Maximum cycle (weeks) with threshold exceeded
Bazett	450 ms 500 ms	*N* = 30 *N* = 7	7.90 (47.40 weeks) 24.14 (144.84 weeks)	2 (12 weeks) 9 (54 weeks)	35 (210 weeks) 33 (198 weeks)
Fridericia	450 ms 500 ms	*N* = 23 *N* = 6	10.39 (62.34 weeks) 23.17 (139.02 weeks)	2 (12 weeks) 9 (54 weeks)	35 (210 weeks) 29 (174 weeks)

In patients with baseline QTc within the normal range (450 ms or lower), we additionally assessed within-patient QTc dynamics over time by calculating delta QTc relative to baseline. Using Bazett’s correction, 52 patients had baseline QTc within the normal range; in 5 of these patients QTc did not increase relative to baseline at any on-treatment assessment, while in the remaining 47 patients the first QTc increase occurred at a mean of cycle 5.21 (range, cycle 2 to 34). The maximum QTc increase relative to baseline was observed later, at a mean of cycle 11.51 (range, cycle 2 to 35). Using Fridericia’s correction, 60 patients had baseline QTc within the normal range; QTc did not increase relative to baseline in 10 patients, while in the remaining 50 patients the first QTc increase occurred at a mean of cycle 6.18 (range, cycle 2 to 34). The maximum QTc increase relative to baseline occurred at a mean of cycle 13.76 (range, cycle 2 to 77).

QTc prolongation was observed not only during the initial phases of sunitinib therapy but also in later treatment cycles. According to both the Bazett and Fridericia correction formulas, the latest occurrence of QTc prolongation meeting grade 1 criteria was noted in cycle 73, grade 2 in cycle 57 and grade 3 in cycle 33.

**Fig. 2:**
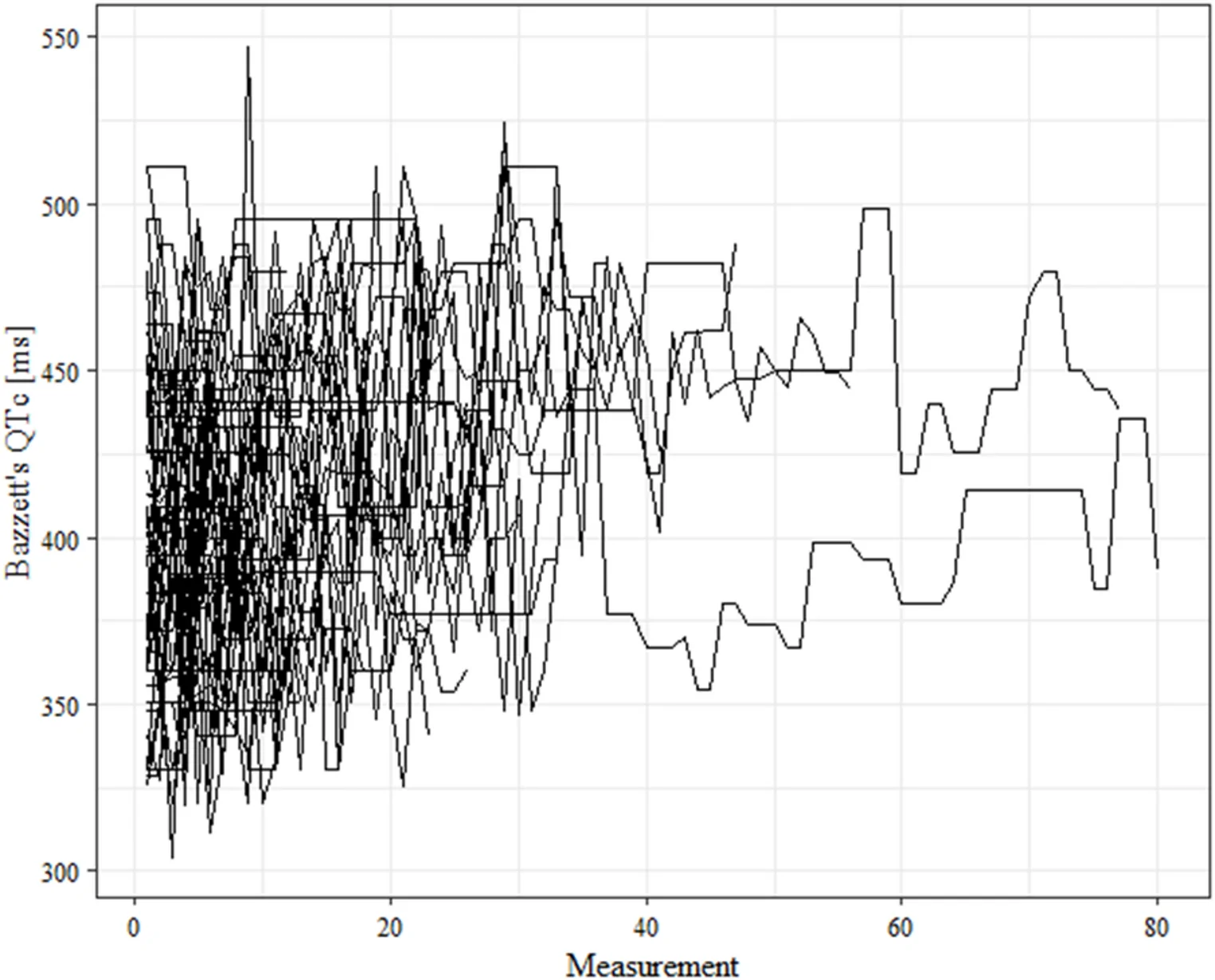
Individual longitudinal trajectories of QTc during treatment across consecutive ECG measurements - QTc corrected with Bazett’s formula

**Fig. 3:**
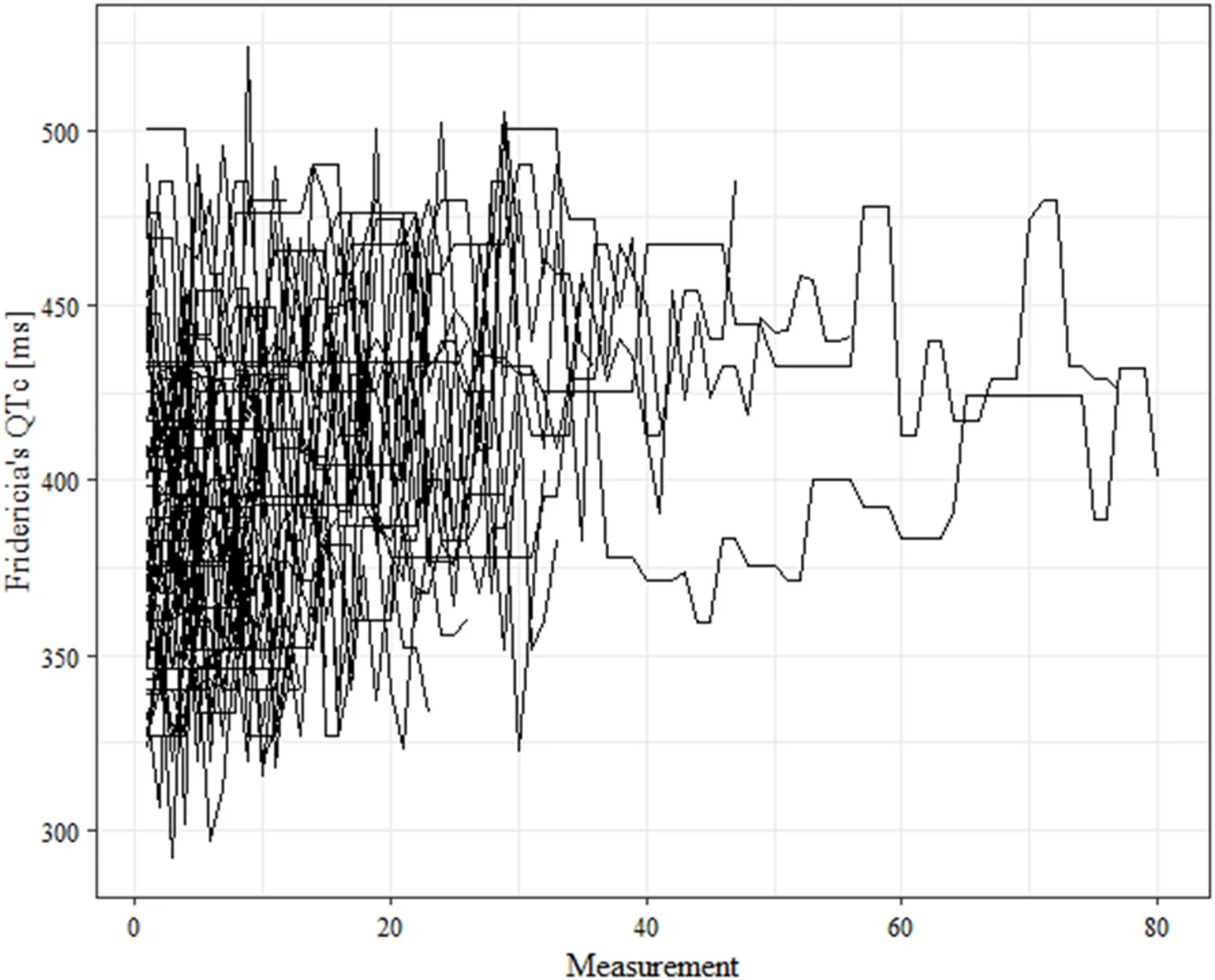
Individual longitudinal trajectories of QTc during treatment across consecutive ECG measurements - QTc corrected with Friderica’s formula

Figures 2 and 3 are longitudinal spaghetti plots showing QTc changes over serial ECG measurements during treatment, calculated using both Bazett’s and Fridericia’s correction.

### Clinical factors associated with QTc prolongation

The analysis of clinical variables potentially associated with QTc interval prolongation identified associations between selected clinical variables and QTc prolongation, stratified by grade and correction formula.

According to Bazett’s formula, 93.3% of patients with QTc prolongation Grade 1 had at least one comorbidity, most frequently arterial hypertension (73.33%), followed by vascular diseases (33.3%), diabetes mellitus and hypothyroidism (23.3% each). A similar pattern was observed for QTc Grade 1 calculated with Fridericia’s formula, where 91.3% had comorbidities and the most frequent was also hypertension (86.9%). For Bazett’s correction, diabetes mellitus and vascular disease remained frequent among patients at Grade 2 (40.00% and 33.33%) and Grade 3 (42.86% and 42.86%). With Fridericia’s correction, the corresponding values were observed in 33.33% and 16.67% of patient, respectively, at both Grade 2 and Grade 3.

In univariate analyses, age and selected comorbidities were the principal correlates of QTc prolongation. For Grade 1, risk rose with higher age at treatment start and with hypothyroidism using Bazett’s correction (OR 1.074 per year, *p* = 0.025; OR 5.326, *p* = 0.048) and with older age at diagnosis and at treatment start and with hypertension using Fridericia’s correction (OR 1.084, *p* = 0.013; OR 1.110, *p* = 0.004; OR 4.198, *p* = 0.038). For Grade 2, Bazett’s correction identified higher age at treatment initiation, and endocrine comorbidities as associated factors (OR 1.077, *p* = 0.038; OR 1.091, *p* = 0.021; OR 6.019, *p* = 0.026). Using Fridericia’s correction Grade 2 QTc prolongation was associated with older age, smoking status, and the presence of soft-tissue metastases (OR 1.116, *p* = 0.011; OR 1.157, *p* = 0.002; OR 5.25, *p* = 0.022; OR 4.471, *p* = 0.027). For Grade 3, only Bazett’s correction was identified significant predictors: older age, smoking and soft-tissue metastases (OR 1.195, *p* = 0.006; OR 1.191, *p* = 0.006; OR 9.652, *p* = 0.042; OR 12.947, *p* = 0.022). No clinical variables were significantly associated with Grade 3 QTc prolongation when Fridericia’s correction was applied.

We did not demonstrate that sex had a significant effect on QTc prolongation calculated using the Bazett’s (G1:OR 0.628, *p* = 0.36; G2:OR 0.425, *p* = 0.151; G3:OR 0.772, *p* = 0.749) or Fridericia’s (G1:OR 0.509, *p* = 0.201; G2:OR 0.528, *p* = 0.32; G3:OR 1.211, *p* = 0.833) formulas.

Other evaluated factors, including body mass index, Karnofsky performance status, MSKCC and IMDC risk scores, sunitinib dose, treatment duration, best response, and treatment-related adverse events, were not significantly associated with QTc prolongation.

## Discussion

In this cohort of patients with advanced ccRCC receiving sunitinib in the first-line setting, QTc prolongation was observed in a notable proportion. This data is consistent with previous reports [10, 12, 24, 27–29]. Importantly, QTc prolongation in our cohort was not confined to the early phase of treatment. In several patients, QTc increases were observed after multiple treatment cycles, including rare late occurrences, suggesting that QTc prolongation may develop at any point during therapy. In the literature, there is no clear evidence that prolongation of the QTc interval is associated with treatment duration [30]. In our cohort, this effect may reflect cumulative exposure to the drug in the case of the 4/2 dosing schedule (consisting of 4 consecutive weeks of daily dosing followed by a 2-week off-treatment interval), as well as comorbidities, concomitant medications, and treatment-related adverse events such as thyroid dysfunction or electrolyte abnormalities, all of which are known to influence cardiac repolarization [10–12, 27–30]. An alternative dosing schedule for sunitinib − 2 weeks on/1 week off – has been reported to improve tolerability [31, 32].

Although it was not used in our group due to reimbursement restrictions at the time, but it could be a practical option in clinical practice.

Several patients in our cohort experienced grade 3 QTc prolongation according to CTCAE v5 criteria. While events with QTc ≥ 500 ms were uncommon, their identification is clinically meaningful, given the association between marked QTc prolongation and an increased risk of potentially life-threatening ventricular arrhythmias. Regulatory and clinical data suggest that sunitinib-related life-threatening arrhythmias are rare but possible [10, 12, 24, 29], underscoring the importance of systematic QTc monitoring and timely risk stratification in cardio-oncology practice.

A key methodological finding of this study is the substantial impact of the QTc correction formula on the classification of QTc prolongation severity. The CTCAE v5 criteria do not specify a preferred QTc correction method [25]; therefore, Bazett’s, Fridericia’s or Framingham’s are commonly used in oncology research and clinical practice [8, 9, 12]. In our cohort, the choice of QTc correction method had a substantial effect on the classification of prolongation severity. Using Bazett’s formula resulted consistently higher QTc values than Fridericia’s correction, which translated into a greater number of patients meeting grade 2 or 3 CTCAE thresholds. This finding is clinically relevant as it mirrors prior cardio-oncology reports demonstrating that Bazett’s correction tends to overestimate QTc at higher HR and underestimate it at lower rates [9, 12], potentially leading to unnecessary treatment interruptions or dose reductions [9]. In oncology populations, deviations in HR are frequent due to anemia, dehydration, infection, fever or medications used [9, 10, 33]. Discontinuing or reducing the dose of sunitinib may decrease its effectiveness in advanced ccRCC treatment [34]. Our findings support current cardio-oncology recommendations favoring Fridericia’s correction over Bazett’s in both clinical practice and research settings [8, 12, 24, 28]. The choice of QTc correction method should not be regarded as minor technical detail but a determinant of treatment continuity in real-world practice.

We identified several clinical factors associated with QTc prolongation, including older age, thyroid dysfunction, arterial hypertension, smoking history, and the presence of soft-tissue metastases. Similar risk factors have been reported in other studies and include pre-existing cardiovascular disease, thyroid abnormalities, electrolyte disturbances, and polypharmacy with QT-prolonging agents or CYP3A4 inhibitors [7, 12, 33, 35–37]. Hypothyroidism induced by TKIs is common and may contribute to QTc prolongation; appropriate management of thyroid function should be part of risk mitigation strategies [27]. It has been shown that hypertension, a significant health problem and the most common complication of TKI therapy, increases the risk of QTc prolongation. Regular monitoring of blood pressure values is necessary [38]. The association between soft-tissue metastases and QTc prolongation observed in our cohort is hypothesis-generating and may reflect altered drug distribution or hepatic metabolism, requiring further investigation.

### Study limitations

This study has several limitations. Its retrospective, single-center design introduces selection bias and limits the generalizability of the findings. The relatively small sample size, particularly for grade 3 QTc prolongation events, reduced statistical power and precluded multivariate analyses.

ECGs were obtained on day 1 of each cycle (after the 14-day off-treatment period). This timing may not coincide with peak sunitinib exposure during the on-treatment phase and may therefore underestimate maximal on-drug QTc prolongation. In addition, post-treatment ECG follow-up was not standardized. Sunitinib plasma concentrations were not measured, and no pharmacokinetic analyses were performed; therefore, associations between QTc prolongation and drug exposure remain speculative.

Although laboratory parameters such as electrolytes and thyroid function were routinely assessed and corrected when clinically indicated, the retrospective design precluded formal time-varying analyses of these factors in relation to QTc dynamics. Similarly, while concomitant medications (including CYP3A4 inhibitors and inducers) were recorded, time-dependent analyses were not performed. Heart rate distribution was not reported, and heart rate–stratified analyses were not conducted; therefore, the impact of heart rate on QTc correction performance and between-formula differences could not be quantified directly.

Attrition over time reduced the number of available observations in later cycles, limiting the reliability of comparisons in these cycles. Repeated per-cycle testing without adjustment for multiplicity may have increased the risk of type I error. In addition, the analysis does not model longitudinal within-patient trajectories or account for informative dropout due to treatment discontinuation, limiting inference on overall time trends and between-formula differences across the entire treatment course. The lack of a contemporaneous control group prevents complete separation of sunitinib-related QTc effects from time-dependent changes associated with prolonged follow-up. Finally, the lack of long-term follow-up prevents assessment of the prognostic significance of QTc prolongation and its association with arrhythmic events.

In summary, in this single-center real-world ccRCC cohort on first-line sunitinib, QTc prolongation was common, with grade 3 events. Risk was higher with older age, thyroid dysfunction, hypertension, smoking and soft-tissue metastases. We recommend QTc monitoring with Fridericia’s correction, ECG at baseline and every 3 months (or each cycle when risk factors are present) and correction of modifiable risk factors. These observations support coordinated cardio-oncology care and the use of predefined cardio-oncology pathways to guide dose reduction and, when necessary, treatment cessation in response to QTc prolongation in patients with ccRCC on first-line sunitinib.

## Supplementary Information

Below is the link to the electronic supplementary material.

Supplementary Material 1

## Data Availability

No datasets were generated or analysed during the current study.
