# Correction: Evaluation of QTc interval prolongation in patients with advanced clear cell renal cell carcinoma treated with first line sunitinib

**DOI:** 10.1007/s00280-026-04945-2

**Published:** 2026-09-19

**Authors:** Agata Sałek-Zań, Mirosława Püsküllüoğlu, Agnieszka Stąpór, Justyna Jaworska, Agnieszka Pietruszka, Aleksandra Grela-Wojewoda, Maciej Stąpór, Tomasz Banaś

**Affiliations:** 1 Department of Clinical Oncology, Kraków Branch, Maria Sklodowska-Curie National Research Institute of Oncology, Garncarska 11, Kraków, 31-115 Poland; 2 Faculty of Medicine, Institute of Cardiology, Department of Interventional Cardiology, Jagiellonian University Medical College, St. John Paul II Hospital, Kraków, Poland; 3 Department of Radiation Oncology, Kraków Branch, Maria Sklodowska-Curie National Research Institute of Oncology, Kraków, Poland

**Correction:**

**Cancer Chemother Pharmacol (2026) 96:40**

https://doi.org/10.1007/s00280-026-04884-y

In this article, the authors name ‘Mirosława Püsküllüoğlu, Agnieszka Stąpór, Justyna Jaworska, Agnieszka Pietruszka, Aleksandra Grela-Wojewoda, Maciej Stąpór, Tomasz Banaś’ were incorrectly written as ‘Püsküllüoğlu Mirosława, Stąpór Agnieszka, Jaworska Justyna, Pietruszka Agnieszka, Grela-Wojewoda Aleksandra, Stąpór Maciej, Banaś Tomasz’.

The original article has been corrected.

