# Supplementary material for: Evaluation of QTc interval prolongation in patients with advanced clear cell renal cell carcinoma treated with first line sunitinib
Source: Cancer Chemother Pharmacol. 2026 Apr 29;96(1):40. doi: 10.1007/s00280-026-04884-y (PMC13128685; doi:10.1007/s00280-026-04884-y)
Supplement: Supplementary file 1 — Supplementary Material 1 [file 280_2026_4884_MOESM1_ESM.docx]

**Table S1.** Comparison of QTc interval values calculated using Bazett’s and Fridericia’s correction formulas across consecutive treatment measurements (weeks/ before number of cycle), (Wilcoxon paired test, p-values shown for each timepoint). Cycle-level QTc summaries are presented up to cycle 40; beyond cycle 40 the number of patients with available ECGs was very small, therefore results are not shown.

| **Parameter** | **Measurement** | **N** | **Mean** | **SD** | **Median** | **Min** | **Max** | **Q1** | **Q3** | **p** |
| --- | --- | --- | --- | --- | --- | --- | --- | --- | --- | --- |
| Baseline  (0 week/ before 1 cycle) | Bazett | 67 | 406,11 | 47,44 | 401,89 | 325,29 | 511,21 | 367,73 | 440,00 | p<0.001 * |
|  | Fridericia | 67 | 395,05 | 45,12 | 389,51 | 323,52 | 500,45 | 356,10 | 432,27 |  |
| Measurement 2  (6 weeks/before 2 cycles) | Bazett | 31 | 407,79 | 42,28 | 408,97 | 326,99 | 487,93 | 378,10 | 441,20 | p<0.001 * |
|  | Fridericia | 31 | 394,23 | 41,03 | 392,90 | 306,77 | 485,28 | 368,42 | 425,06 |  |
| Measurement 3  (12 weeks/before 3 cycles) | Bazett | 33 | 391,78 | 38,74 | 400,00 | 303,87 | 450,19 | 360,15 | 417,42 | p<0.001 * |
|  | Fridericia | 33 | 381,16 | 37,24 | 392,24 | 292,14 | 432,79 | 357,98 | 409,02 |  |
| Measurement 4  (18 weeks/befor 4 cycles) | Bazett | 33 | 395,15 | 44,82 | 386,44 | 319,47 | 484,42 | 360,00 | 435,98 | p<0.001 * |
|  | Fridericia | 33 | 384,87 | 41,27 | 382,56 | 302,05 | 467,57 | 351,82 | 415,13 |  |
| Measurement 5  (24 weeks/befor 5 cycles) | Bazett | 37 | 405,85 | 44,63 | 396,25 | 320,00 | 495,74 | 378,63 | 438,18 | p<0.001 * |
|  | Fridericia | 37 | 393,80 | 43,54 | 386,07 | 320,00 | 490,44 | 363,57 | 425,06 |  |
| Measurement 6  (30 weeks/before 6 cycles) | Bazett | 30 | 407,59 | 44,16 | 406,29 | 311,77 | 480,00 | 383,25 | 443,53 | p<0.001 * |
|  | Fridericia | 30 | 393,59 | 42,99 | 395,84 | 297,17 | 480,00 | 366,52 | 424,79 |  |
| Measurement 7  (36 weeks/before 7 cycles) | Bazett | 32 | 396,36 | 42,56 | 398,02 | 328,63 | 484,09 | 369,50 | 425,27 | p<0.001 * |
|  | Fridericia | 32 | 386,08 | 42,16 | 383,72 | 311,35 | 495,78 | 352,21 | 419,05 |  |
| Measurement 8  (42 weeks/before 8 cycles) | Bazett | 29 | 407,84 | 38,67 | 402,13 | 342,54 | 495,20 | 386,44 | 419,52 | p=0.001 * |
|  | Fridericia | 29 | 399,27 | 37,76 | 390,91 | 351,82 | 485,28 | 367,83 | 412,91 |  |
| Measurement 9  (48 weeks/before 9 cycles) | Bazett | 30 | 405,04 | 45,71 | 400,00 | 320,00 | 547,28 | 379,78 | 433,44 | p=0.001 * |
|  | Fridericia | 30 | 395,75 | 45,38 | 398,12 | 320,00 | 523,87 | 363,57 | 423,67 |  |
| Measurement 10  (54 weeks/before 10 cycles) | Bazett | 28 | 402,95 | 40,69 | 402,58 | 320,00 | 480,00 | 377,57 | 430,76 | p<0.001 * |
|  | Fridericia | 28 | 392,39 | 41,29 | 394,73 | 315,79 | 480,00 | 369,61 | 418,59 |  |
| Measurement 11  (60 weeks/before 11 cycles) | Bazett | 25 | 408,69 | 45,80 | 405,17 | 333,07 | 491,93 | 371,81 | 450,19 | p<0.001 * |
|  | Fridericia | 25 | 395,88 | 46,76 | 389,51 | 317,97 | 489,34 | 357,98 | 432,79 |  |
| Measurement 12  (66 weeks/ before 12 cycles) | Bazett | 22 | 411,57 | 41,16 | 405,17 | 350,54 | 480,00 | 371,79 | 447,18 | p=0.002 * |
|  | Fridericia | 22 | 402,06 | 44,98 | 393,21 | 340,05 | 480,00 | 359,47 | 432,79 |  |
| Measurement 13  (72 weeks/before 13 cycles) | Bazett | 23 | 408,98 | 42,46 | 413,12 | 330,49 | 482,00 | 376,14 | 443,08 | p=0.001 * |
|  | Fridericia | 23 | 398,74 | 41,00 | 397,23 | 326,96 | 469,09 | 368,78 | 431,40 |  |
| Measurement 14  (78 weeks/before 14 cycles) | Bazett | 17 | 429,25 | 43,03 | 435,12 | 347,79 | 495,74 | 405,17 | 452,55 | p=0.005 * |
|  | Fridericia | 17 | 418,02 | 40,69 | 418,11 | 351,82 | 490,44 | 389,51 | 440,26 |  |
| Measurement 15  (84 weeks/before 15 cycles) | Bazzett | 19 | 421,29 | 45,57 | 420,86 | 330,49 | 484,42 | 395,72 | 458,16 | p=0.001 * |
|  | Fridericia | 19 | 410,21 | 41,48 | 408,70 | 326,96 | 480,00 | 383,44 | 443,87 |  |
| Measurement 16  (90 weeks/before 16 cycles) | Bazett | 17 | 420,08 | 44,30 | 425,08 | 330,49 | 495,20 | 386,44 | 444,22 | p=0.001 * |
|  | Fridericia | 17 | 411,24 | 42,60 | 417,04 | 326,96 | 476,07 | 387,07 | 440,00 |  |
| Measurement 17  (96 weeks/before 17 cycles) | Bazett | 16 | 415,48 | 45,96 | 415,12 | 350,54 | 495,20 | 377,07 | 444,00 | p=0.028 * |
|  | Fridericia | 16 | 409,15 | 44,93 | 408,52 | 340,05 | 476,07 | 376,03 | 452,32 |  |
| Measurement 18  (102 weeks/before 18 cycles) | Bazett | 15 | 421,87 | 35,91 | 425,83 | 360,00 | 482,00 | 403,41 | 447,20 | p=0.021 * |
|  | Fridericia | 15 | 415,19 | 34,47 | 417,04 | 360,00 | 467,57 | 391,21 | 436,40 |  |
| Measurement 19  (108 weeks/before 19 cycles) | Bazett | 14 | 427,54 | 45,38 | 425,01 | 345,64 | 511,00 | 399,80 | 460,25 | p=0.043 * |
|  | Fridericia | 14 | 421,87 | 46,92 | 424,94 | 336,87 | 500,45 | 386,07 | 457,64 |  |
| Measurement 20  (116 weeks/before 20 cycles) | Bazett | 13 | 411,69 | 41,51 | 400,00 | 350,54 | 471,93 | 383,25 | 450,19 | p=0.018 * |
|  | Fridericia | 13 | 405,00 | 40,55 | 400,00 | 340,05 | 474,61 | 377,88 | 435,06 |  |
| Measurement 21  (122 weeks/before 21 cycles) | Bazett | 13 | 421,17 | 53,79 | 415,69 | 325,29 | 511,21 | 379,47 | 468,42 | p=0.014 * |
|  | Fridericia | 13 | 410,47 | 49,23 | 396,23 | 323,52 | 476,07 | 382,56 | 458,75 |  |
| Measurement 22  (128 weeks/before 22 cycles) | Bazett | 12 | 433,51 | 51,72 | 439,09 | 360,00 | 495,31 | 383,50 | 485,30 | p=0.014 * |
|  | Fridericia | 12 | 423,00 | 43,23 | 428,93 | 360,00 | 476,07 | 385,61 | 462,83 |  |
| Measurement 23  (134 weeks/before 23 cycles) | Bazett | 15 | 421,19 | 42,40 | 413,12 | 340,67 | 480,00 | 391,62 | 456,95 | p=0.017 * |
|  | Fridericia | 15 | 413,80 | 43,19 | 408,70 | 333,63 | 480,00 | 379,11 | 451,79 |  |
| Measurement 24  (140 weeks/before 24 cycles ) | Bazett | 11 | 430,14 | 42,48 | 440,00 | 353,95 | 493,32 | 394,36 | 455,72 | p=0.096 |
|  | Fridericia | 11 | 424,67 | 46,02 | 432,79 | 355,95 | 502,05 | 382,56 | 451,72 |  |
| Measurement 25  (146 weeks/before 25 cycles) | Bazett | 10 | 416,71 | 40,04 | 397,18 | 365,95 | 482,00 | 394,36 | 447,09 | p=0.024 * |
|  | Fridericia | 10 | 408,16 | 37,02 | 391,28 | 363,96 | 467,57 | 382,56 | 443,11 |  |
| Measurement 26  (152 weeks/before 26 cycles) | Bazett | 10 | 417,62 | 26,35 | 422,78 | 360,00 | 447,27 | 407,57 | 437,08 | p=0.014 * |
|  | Fridericia | 10 | 409,93 | 27,23 | 414,28 | 360,00 | 444,84 | 393,73 | 428,12 |  |
| Measurement 27  (158 weeks/before 27 cycles) | Bazett | 11 | 437,32 | 31,93 | 440,00 | 371,81 | 482,00 | 425,65 | 448,68 | p=0.009 * |
|  | Fridericia | 11 | 426,09 | 30,33 | 432,79 | 367,83 | 467,57 | 410,40 | 440,00 |  |
| Measurement 28  (164 weeks/28 cycles) | Bazett | 9 | 433,73 | 42,91 | 432,60 | 371,81 | 487,93 | 399,80 | 480,00 | p=0.08 |
|  | Fridericia | 9 | 427,46 | 42,88 | 425,06 | 367,83 | 485,28 | 390,91 | 467,57 |  |
| Measurement 29  (170 weeks/before 29 cycles) | Bazett | 8 | 455,82 | 60,35 | 445,47 | 347,79 | 524,48 | 432,56 | 512,86 | p=0.016 * |
|  | Fridericia | 8 | 442,78 | 55,81 | 430,66 | 351,82 | 505,31 | 417,86 | 500,85 |  |
| Measurement 30  (176 weeks/before 30 cycles) | Bazett | 10 | 439,39 | 43,75 | 445,09 | 346,72 | 495,74 | 419,34 | 472,55 | p=0.107 |
|  | Fridericia | 10 | 432,57 | 46,87 | 432,79 | 322,88 | 490,44 | 426,11 | 460,68 |  |
| Measurement 31  (182 weeks/before 31 cycles) | Bazett | 5 | 409,01 | 41,76 | 419,52 | 347,79 | 450,19 | 387,54 | 440,00 | p=0.201 |
|  | Fridericia | 5 | 400,22 | 40,28 | 412,91 | 351,82 | 440,00 | 363,57 | 432,79 |  |
| Measurement 32  (188 weeks/before 32 cycles) | Bazett | 8 | 432,14 | 39,21 | 437,08 | 360,00 | 475,25 | 417,79 | 462,17 | p=0.035 * |
|  | Fridericia | 8 | 417,28 | 33,74 | 416,49 | 360,00 | 463,20 | 400,92 | 433,48 |  |
| Measurement 33  (194 weeks/before 33 cycles) | Bazett | 7 | 444,77 | 51,47 | 438,18 | 369,50 | 511,21 | 415,17 | 482,08 | p=0.016 * |
|  | Fridericia | 7 | 427,01 | 49,78 | 425,06 | 352,21 | 490,44 | 395,79 | 464,91 |  |
| Measurement 34  (200 weeks/ before 34 cycles) | Bazett | 6 | 446,90 | 12,63 | 444,22 | 438,18 | 471,93 | 439,69 | 444,56 | p=0.058 |
|  | Fridericia | 6 | 434,72 | 19,63 | 427,30 | 425,06 | 474,61 | 425,21 | 428,96 |  |
| Measurement 35  (206 weeks/before 35 cycles) | Bazett | 5 | 430,23 | 50,20 | 456,07 | 360,15 | 472,14 | 394,36 | 468,42 | p=0.062 |
|  | Fridericia | 5 | 416,42 | 50,00 | 436,56 | 346,23 | 458,75 | 382,56 | 458,01 |  |
| Measurement 37  (218 weeks/before 37 cycles) | Bazett | 5 | 422,04 | 52,04 | 438,60 | 360,15 | 484,42 | 376,82 | 450,19 | p=0.125 |
|  | Fridericia | 5 | 407,98 | 44,49 | 428,51 | 346,23 | 454,47 | 377,88 | 432,79 |  |
| Measurement 40  (236 weeks/before 40 cyles) | Bazett | 5 | 429,24 | 43,04 | 423,16 | 367,11 | 482,00 | 419,52 | 454,43 | p=0.125 |
|  | Fridericia | 5 | 422,57 | 37,39 | 412,91 | 371,36 | 467,57 | 411,45 | 449,57 |  |

p - Wilcoxon paired test

* statistically significant (p<0.05)
